# Refined Contact Map Prediction of Peptides Based on GCN and ResNet

**DOI:** 10.3389/fgene.2022.859626

**Published:** 2022-04-27

**Authors:** Jiawei Gu, Tianhao Zhang, Chunguo Wu, Yanchun Liang, Xiaohu Shi

**Affiliations:** ^1^ College of Computer Science and Technology, University of Jilin, Changchun, China; ^2^ Key Laboratory of Symbolic Computation and Knowledge Engineering, Ministry of Education, Changchun, China; ^3^ School of Computer Science, Zhuhai College of Science and Technology, Zhuhai, China

**Keywords:** peptide inter-residue contact map prediction, deep learning, graph convolutional network, residual convolutional neural network, multiple sequence alignment

## Abstract

Predicting peptide inter-residue contact maps plays an important role in computational biology, which determines the topology of the peptide structure. However, due to the limited number of known homologous structures, there is still much room for inter-residue contact map prediction. Current models are not sufficient for capturing the high accuracy relationship between the residues, especially for those with a long-range distance. In this article, we developed a novel deep neural network framework to refine the rough contact map produced by the existing methods. The rough contact map is used to construct the residue graph that is processed by the graph convolutional neural network (GCN). GCN can better capture the global information and is therefore used to grasp the long-range contact relationship. The residual convolutional neural network is also applied in the framework for learning local information. We conducted the experiments on four different test datasets, and the inter-residue long-range contact map prediction accuracy demonstrates the effectiveness of our proposed method.

## 1 Introduction

Peptides play an important role in computational and experimental biology (Torrisi et al., 2020), which motivates the development of accurate methods to predict their native conformations from the sequences. As a special kind of peptide, protein-related predictions from its amino acid sequence remain an open problem in the field of computational biology. Using biological experiments to determine the protein structure is very cumbersome and expensive. Therefore, it is very effective to use machine learning methods or deep learning methods to obtain a universal law from the amino acid sequence to the prediction of a protein’s three-dimensional structure. The inter-residue contact map (Lena et al., 2012) is a two-dimensional representation of a protein’s three-dimensional structure. The contact map constrains the conformation of protein structures; as a result, accurate prediction of the contact map can facilitate *ab initio* structure modeling, and the accuracy of the contact map affects the accuracy of the three-dimensional structure of the protein. Furthermore, contact maps have been widely used for model assessment and structure alignment.

The current contact map prediction methods are mainly based on direct coupling analysis (DCA) methods, machine learning methods, and deep learning methods. DCA-based methods mainly use multiple sequence alignment methods to determine the relationships between amino acid pairs. However, DCA-based methods assume that pairs of contacted residues are more likely to mutate simultaneously as the protein structure or function evolves and mainly use the multiple sequence alignment (MSA) to determine the relationships between the amino acid pairs. Therefore, the accuracy of the DCA-based method depends on the number of homologous protein sequences in the protein sequence library. On the other hand, due to the existence of indirect evolutionary coupling information, the generated coupling information from the DCA might include “noise signal.” The common DCA-based methods include CCMpred (Seemayer et al., 2014), PSICOV (Jones et al., 2012), and GREMLIN (Kamisetty et al., 2013). CCMpred mainly uses Markov random field pseudo-likelihood maximization to learn the contacts between the protein inter-residues. When there are a large number of homologous proteins in the protein sequence, the accuracy of the contact prediction results is higher; however, when the sequences of the homologous protein are fewer, the accuracy is lower. On the other hand, machine learning-based and deep learning-based methods use a set of input features derived from multiple sequence alignments (MSAs) to predict the protein inter-residue contact map, including position-specific scoring matrices (PSSMs), secondary structure (SS) predictions, and solvent accessibility (SA) information. Machine learning-based methods are mainly based on support vector machines (SVMs) (Hearst et al., 1998) to learn the abovementioned features and common support vector machine (SVM) methods including SVMCon (Cheng and Baldi, 2007) and R2C (Yang et al., 2016). SVMCon used support vector machines (SVMs) and yields good performance on medium- to long-range contact predictions. In recent years, deep learning methods have been mainly used to predict the contact map between the protein inter-residues and are mainly based on the structure of the convolutional neural network (CNN) and residual neural network (ResNet) (He et al., 2016). The ResNet structure further improves the CNN structure and solves the problem of reduced accuracy when there are too many convolutional layers through the skip connection mechanism. RaptorX-Contact (Wang et al., 2017) was the first model that used the ResNet structure for protein inter-residue contact map prediction tasks. Zhong Li et al. (Li et al., 2020) used ResNet and DenseNet (Huang et al., 2017) structures and a new protein sequence feature (PSFM) to improve the contact map prediction accuracy. DeepCov (Jones and Kandathil, 2018) applied the CNN to predict contact maps when limited evolutionary information is available, which has been trained on a very limited set of input features: pair frequencies and covariance. It is noticed that there are several similar studies predicting the distance matrix instead of the contact map, such as RaptorX structure prediction (Xu, 2018), PG-GNN (Xia and Ku, 2020), and AlphaFold (Senior et al., 2020).

However, there are two main difficulties in obtaining accurate contact predictions. First, many amino acid sequences lack a large number of homologous sequences, which limits the level of accuracy of predictions. On the contrary, the target sequences with many homologous sequences might generate “noise signals” from the evolutionary coupling information. Second, most methods use convolutional neural network (CNN)-based models for inter-residue contact map prediction, leading to over-learning of the local information, but under-learning of the long-range information, which is reflected by a low long-range accuracy.

Therefore, eliminating “noise signals” is necessary to improve the residue contact prediction. Improving the inter-residue contact prediction has been of interest for many years due to its critical importance in structure bioinformatics, with either the sequence or structure template information. R2C (Yang et al., 2016) used SVM and PSICOV methods and used a dynamic fusion strategy to predict the contact map between amino acids and applied Gaussian noise filters for further denoising. Amelia Villegas-Morcillo et al. (Villegas-Morcillo et al., 2018) applied K-SVD (Aharon et al., 2006) and deep convolutional neural network (DCNN) methods specially designed for image denoising to solve the problem of Gaussian noise. DNCON2 (Adhikari et al., 2017) adopted the structure of the two-stage convolutional neural networks (CNNs) to improve the contact map prediction, which divides the prediction into two parts. The first part trains five CNNs to predict the contact map between the distances of 6, 7.5, 8, 8.5, and 10, respectively. The second part takes the input feature as the output of the first part and then utilizes a CNN structure for further prediction.

In the past few years, the graph neural network (Zhou et al., 2018) was raised to represent the protein structure in various deep learning-based methods and had succeeded in the computational biology area, such as protein interface prediction, protein solubility prediction, and protein function prediction. Fout et al., (2017) proposed a type of architecture for the task of predicting protein interfaces between the pairs of proteins using a graph representation of the underlying protein structure. GraphSol (Chen et al., 2020) was used to predict the protein residue solubility by combining the predicted contact maps, graph neural networks, and attention mechanisms. DeepFRI (Gligorijević et al., 2021) used an LSTM (Hochreiter and Schmidhuber, 1997) and a graph convolutional network to predict protein functions. PG-GNN (Xia and Ku, 2020) used a new convolution kernel to perform deep convolution to obtain the distance map, which was used to construct an inter-residue graph between the residues for obtaining the dihedral information between residues, and finally constructed a three-dimensional protein structure.

Here, to focus on getting more accurate contact maps, especially on the long-range level, we developed a novel refined contact map prediction model (RCMPM) to refine the rough contact map produced by the existing methods, which combines a graph convolution network (GCN) (Kipf and Welling, 2016) and residual convolution neural networks (ResNet) (He et al., 2016). The main contributions of the article are summarized as follows:

• The peptide contact map refinement task is modeled as a geometric 2D graph improvement, with nodes representing the amino acid residues and edges representing contacts between the residues. The rough results of other models such as CCMpred and RaptorX-Contact are used to construct the inter-residue contact graph.
• Aiming at the challenges previously mentioned, a novel deep neural network framework is proposed for the inter-residue contact prediction by combining a graph convolution network (GCN) and residual convolution neural networks (1D ResNet and 2D ResNet), of which the GCN has a strong global information extraction ability, and hence can better capture the long-range contact relationships among the complex sequence inter-residues.
• The experiments are conducted on four different test datasets, and the inter-residue long-range contact map prediction accuracy demonstrates the effectiveness of our proposed method due to the new network architecture.

The rest of the article is organized as follows. Section 2 details the materials and methods, including contact definition, graph construction, feature selection, and the proposed prediction model. Section 3 reports the datasets used in our method, evaluation metrics, and experiments on four test datasets. Section 4 concludes the article and discusses the directions for the future work.

## 2 Materials and Methods

### 2.1 Contact Definition

In general, two residues are considered to be in contact if certain atoms are close enough to form a molecular interaction. In the Critical Assessment of protein Structure Prediction (CASP) experiment (Moult et al., 2014, 2016, 2018), the contact definition is based on the spatial distance of *C*_*β*_ atoms. For instance, assuming that $v=\left\{v_{1},v_{2},\ldots ,v_{i},\ldots ,v_{j},\ldots ,v_{L}\right\}$ is the residue sequence, where *L* is the sequence length, and $\left(x_{v_{i}},y_{v_{i}},z_{v_{i}}\right)$ is the three-dimensional coordinates of amino acid residue *v*_*i*_, then the equation for the distance between the residues *v*_*i*_ and *v*_*j*_ is

$$\begin{array}{cc} Distance\left(i,j\right) & =Distance\left(C_{\beta_{i}},C_{\beta_{j}}\right)\\ & =\sqrt{{\left(x_{v_{i}}-x_{v_{j}}\right)}^{2}+{\left(y_{v_{i}}-y_{v_{j}}\right)}^{2}+{\left(z_{v_{i}}-z_{v_{j}}\right)}^{2}} \end{array}.$$ (1)

If the Euclidean distance between the *C*_*β*_ atoms (*C*_*α*_ for GLY) of two amino acids is less than a given threshold *γ*, then the two residues are said to be in contact.

### 2.2 Graph Construction

As mentioned in Section 2.1, we can use the other contact map prediction models, such as CCMpred (Seemayer et al., 2014) and RaptorX-Contact (Wang et al., 2017), to obtain a contact matrix *CM*. Assuming that the length of the peptide is *L*, then *CM* is an *L*×*L* matrix, whose element *CM*_*ij*_ denotes whether the pair of residues *i* and *j* is contacted or not (1 or 0). Denote $G=\left\{N,E\right\}$ is the contact graph of the peptide, where *N* is the node set including *L* amino acids, and *E* is the edge set. Then, the contact graph could be constructed as follows:

Algorithm 1 Graph construction.

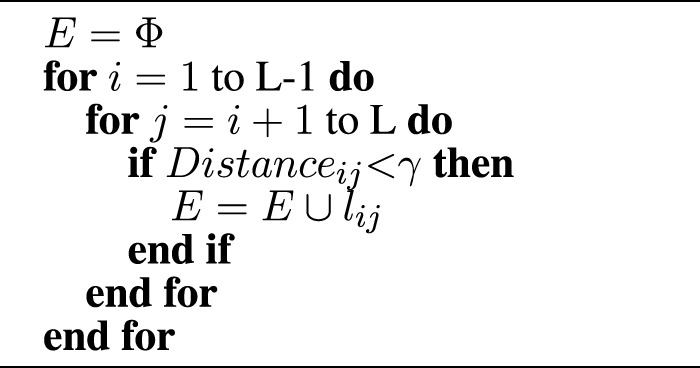

where *l*_*ij*_ is the edge between node *i* and node *j*, and the threshold *γ* is set as 8Å in this article.

### 2.3 Feature Selection

#### 2.3.1 Sequence Features

We devised three groups of sequence features to train our model, namely, the position-specific scoring matrix (PSSM), secondary structure (SS), and solvent accessibility (SA). The PSSM is a widely used sequence feature, which is produced by executing PSI-BLAST (Altschul et al., 1997) on the UniRef90 database (Suzek et al., 2015) with 0.001 e-value after the three iterations, which is a 20-dimensional profile feature for each residue. The secondary structure and solvent accessibility describe the arrangement of the protein backbone, which are also very important for the contact prediction. The secondary structure and solvent accessibility are predicted by the RaptorX-Property (Wang et al., 2016) program (http://raptorx.uchicago.edu/StructurePropertyPred/predict/). The secondary structure is divided into three categories, namely, helix (H), strand (E), and coil (C), and the solvent accessibility is also classified into three types, namely, buried, medium, and exposed. The PSSM is represented as a two-dimensional matrix of *L* × 20, while both the secondary structure and solvent accessibility are represented as a two-dimensional matrix of *L* × 3; therefore, the concatenation sequence embedding vector *X*_*seq*_ is obtained with the *L* × 26 dimension, where the order of the splicing input is [PSSM, secondary structure, and solvent accessibility].

#### 2.3.2 Pairwise Features

Pairwise features are the information that characterizes the relationship between the pairs of residues, including the co-evolutionary information, statistical information, and so on. Four groups of pairwise features are used to train our model, namely, RaptorX-Contact prediction, CCMpred prediction, mutual information (Dunn et al., 2008), and contact potential (Betancourt and Thirumalai, 1999), which provide the co-evolutionary information for each pair of alignment columns. RaptorX-Contact and CCMpred prediction are mainly used as inter-residue scores. RaptorX-Contact prediction results can be obtained by model training, the source code of which can be downloaded from https://github.com/j3xugit/RaptorX-Contact. CCMpred prediction results can be obtained by the CCMpred program, which could be accessed at https://github.com/soedinglab/CCMpred. However, CCMpred requires the homologous sequence result of the multiple sequence alignment (MSA) as the input, which is produced by executing the HHblits program (Remmert et al., 2012) on the Uniclust30 database (Mirdita et al., 2017) with 0.001 e-value after three iterations. Both RaptorX-Contact and CCMpred output an inter-residue score for each residue pair. After the MSA profile is obtained, the mutual information could be defined by

$$MI_{ij}=\underset{x,y\in R}{\sum }p_{ij}\left(x,y\right)\ln\frac{p_{ij}\left(x,y\right)}{p_{i}\left(x\right)p_{j}\left(y\right)},$$ (2)

where *R* is the set of amino acid types, *x* and *y* are the elements in column *i* and column *j*, respectively, *p*_*i*_(*x*) and *p*_*j*_(*y*) indicate the probabilities of residue *x* in column *i* and residue *y* in column *j*, and *p*_*ij*_(*x*, *y*) is the probability that residue *x* is in column *i* and residue *y* is in column *j*, respectively. Normalized mutual information, namely, average product correction (APC) mutual information is also used in our method, which is defined by

$$MI_{ij}^{APC}=MI_{ij}-APC_{ij},$$ (3)

$$APC_{ij}=\frac{\underset{j\neq i}{\sum }MI_{ij}\underset{i\neq j}{\sum }MI_{ij}}{\underset{i,j\left(i\neq j\right)}{\sum }MI_{ij}}.$$ (4)

The contact potential is computed by averaging the contact potential terms across the two alignment columns. Mutual information and contact potential are generated by alnstats in the MetaPSICOV (Jones et al., 2015) program, which also requires the homologous sequence as the input. For RaptorX-Contact prediction, CCMpred prediction, mutual information, APC mutual information, and contact potential, all are represented as a three-dimensional matrix of *L* × *L* × 1; therefore, the concatenation pair-wise embedding features *X*_*pair*_ are obtained with the *L* × *L* × 5 dimension, where the order of the splicing input is [RaptorX-Contact prediction, CCMpred prediction, MI, APC MI, and contact potential].

### 2.4 Prediction Model

#### 2.4.1 The Framework of the RCMPM Model

Residual networks (ResNets) are very helpful for accurate peptide contact map prediction, which has been demonstrated in the RaptorX-Contact model (Wang et al., 2017). Therefore, ResNet architecture is retained in our proposed refined contact map prediction model (RCMPM). On the other hand, the rough contact map obtained by the other methods could be well utilized by transferring it into an amino acid graph, and therefore, the graph convolution network (GCN) could handle the graph topology very well. Hence, the proposed RCMPM model includes a GCN module, a 1D ResNet module, and a 2D ResNet module, respectively.

**FIGURE 1:**
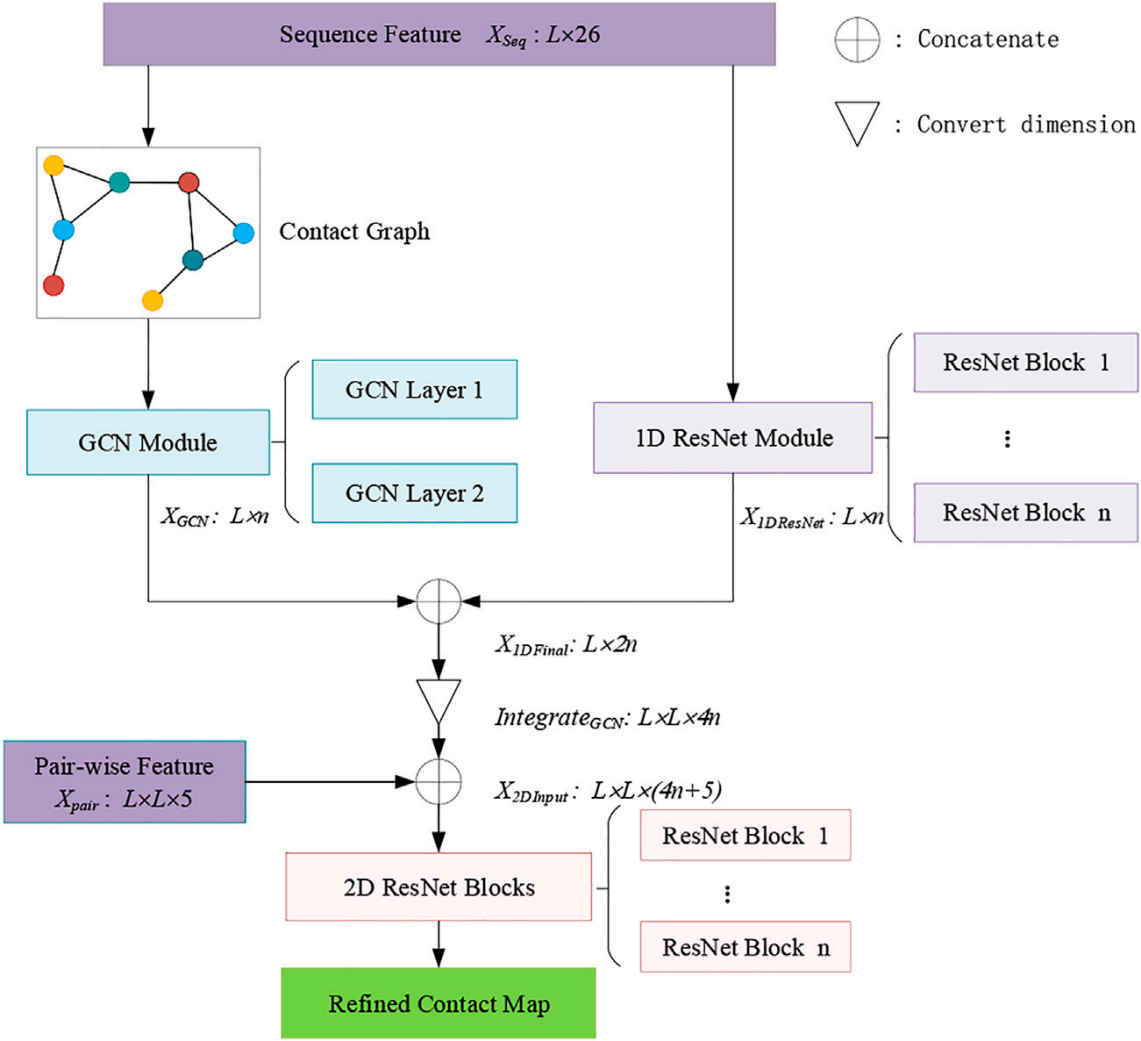
Framework of the RCMPM.

Figure 1 shows the framework of the RCMPM model, which has two types of features, namely, sequence features and pair-wise features. The GCN module is used to learn the global structural features of the inter-residue contact graphs, whose input is the node representation of the sequence features, and the output is a dense global structural embedding vector for each amino acid node. 1D ResNet module is used to handle the one-dimensional sequence feature and output a sequence embedding vector for each amino acid. 2D ResNet module integrates the above two modules’ outputs and the pair-wise features as well and finally generates the refined contact map. The following part of this section will describe these three modules in detail.

#### 2.4.2 GCN Module

Given a sequence with *L* residues, the residue graph can be represented by a contact map, that is, the nodes of the graph are the residues of the peptide, and the features of the nodes are represented by the attributes of the residues. The edges of the contact graph indicate whether there are connections between the amino acid nodes, and the weight of the edge represents the probability of contact. We used the graph convolution network (GCN) to obtain the global structural features of the graph.

The graph convolutional layer in the prediction model uses the following equation:

$$H^{\left(l+1\right)}=\sigma \left(\tilde{A}H^{\left(l\right)}W^{\left(l\right)}\right),$$ (5)

where $\tilde{A}=A+I_{L}$ is the variant of the adjacency matrix by adding the self-loop identity matrix *I*_*L*_ on the original adjacency matrix *A*, and *H* ^(*l*)^ is the hidden matrix learned by the *l*th layer, initial of which is the hidden matrix *H* ^(0)^ = *X*_*seq*_. *W* ^(*l*)^ is a weight matrix of the layer-specific trainable parameters and is used to map the iterations to a low-dimensional rich information space, and *σ* is a nonlinear activation function, which is taken as the ReLU function in our model. We also use normalization to map the input feature of each layer *H* ^(*l*)^ to [0,1] to improve the data performance and reduce errors. Finally, we used a 2-layer graph convolutional network to learn the global structural features of the contact graph containing amino acid node features. Hence, the final output of the GCN module in the RCMPM model uses the following equation:

$$X_{GCN}=RELU\left(\tilde{A}ReLU\left(\tilde{A}X_{seq}W^{\left(0\right)}\right)W^{\left(1\right)}\right).$$ (6)

#### 2.4.3 1D ResNet Module

**FIGURE 2:**
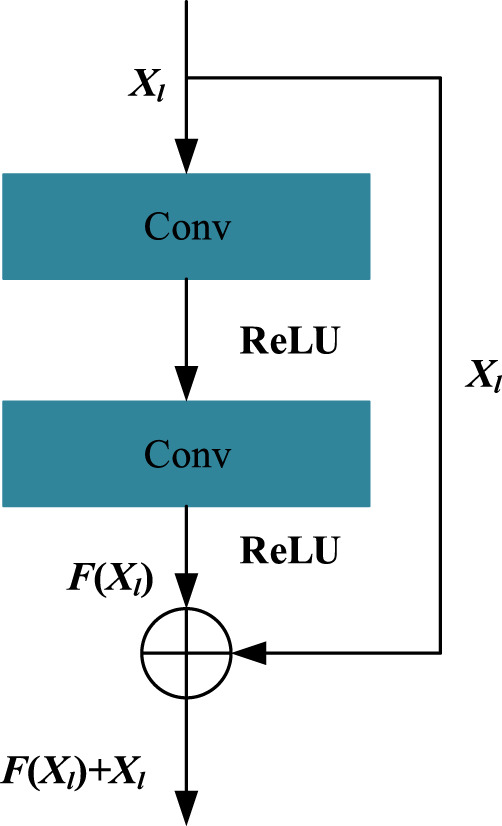
Structure of the residual block.

A 1D ResNet module is used to handle the one-dimensional sequence feature and outputs a sequence embedding vector for each residue, which is stitched together by the residual blocks. A residual block consists of two convolutional layers and two activation layers, which can be defined as follows:

$$X_{l+1}=F\left(X_{l},W_{l}\right)+X_{l},$$ (7)

where *X*_*l*_ and *X*_*l*+1_ are the input and output vectors of the residual block, respectively, and the initial hidden matrix *X*_0_ = *X*_*seq*_. Here, *W*_*l*_ is the weight matrix in convolutional layers of the *l*th block, and *F*(*X*_*l*_, *W*_*l*_) represents the result after the action of the convolutional layer and activation function layer. Here, the operation of the convolutional layer is implemented by the conv1d function of the tensorflow framework. Here, we used the *ReLU* function as the activation function of our method and also used normalization to map the data to [0,1] to improve data performance and reduce errors. We kept the dimension of *X*_*l*+1_ larger than *X*_*l*_ because the higher dimension can carry more information. For a residual block, the *F*(*X*_*l*_, *W*_*l*_) function can be expressed as shown in Figure 2.

Finally, the output of the 1D ResNet module in the RCMPM model could be described as follows:

$$X_{1DResNet}=\overset{n}{\underset{l=0}{\sum }}F\left(X_{l},W_{l}\right)+X_{l}.$$ (8)

In our 1D ResNet module, the number of residual blocks is selected as 3.

#### 2.4.4 2D ResNet Module

The 2D ResNet module is used to learn the final contact relationship for each residue pair by integrating the aforementioned two modules, namely, that it takes the input of the output feature *X*_*GCN*_ of the GCN module and the output feature *X*_*1DResNet*_ of the 1D ResNet module and the pairwise feature *X*_*pair*_ as well. Different with the 1D ResNet module, the 2D ResNet module is dealing with two-dimensional feature maps. The pairwise features *X*_*pair*_ is of *L* × *L* × 5 dimension, as described in section 2.3.2, while the output features *X*_*GCN*_ and *X*_*1DResNet*_ are the one-dimensional feature map with the same dimension *L* × *n*, which should be converted to a two-dimensional feature map. Similarly with the method used in (Wang et al., 2017), *X*_*GCN*_ and *X*_*1DResNet*_ are first concatenated on the second dimension, obtaining an *L* × 2*n* feature map *X*_*1DFinal*_:

$$X_{1DFinal}=X_{1DResNet}⊕X_{GCN}.$$ (9)

Then, it is converted to a 2-dimensional feature map. Redefined *X*_*1DFinal*_ from *L* × 2*n* to *L* × 1 × 2*n* dimension by adding a second-order dimension with 1, then duplicate *X*_*1DFinal*_ *L* times to extend the second order from 1 to *L*, getting an *L* × *L* × 2*n* tensor $TX_{GCN_{1D}}$ . $TX_{GCN}^{\prime }$ is denoted as the transpose of $TX_{GCN_{1D}}$ on the first two orders; *X*_*GCN*_ and *X*_*1DResNet*_ are finally integrated as *Integrate*_*GCN*_:

$$Integrate_{GCN}=TX_{GCN_{1D}}⊕TX_{GCN_{1D}}^{\prime },$$ (10)

where ⊕ represents concatenation on the third-order dimension; therefore $Integrate_{GCN_{1D}}$ is of *L* × *L* × 4*n* dimension. Afterward, it should be combined with the pairwise features *X*_*pair*_ by

$$X_{2DInput}=Integrate_{GCN}⊕X_{pair},$$ (11)

where *X*_*2DInput*_ is of *L* × *L* × (4*n* + 5) dimension finally.

We also used the same residual network block structure with that of the 1D ResNet (Figure 2) module to stack the 2D ResNet module. The difference is that the 2D ResNet module is dealing with 2D feature maps and utilizing *conv*2*d* function of the tensorflow framework for the convolution operation. The final output *X*_*2DResNet*_ of the 2D ResNet module could be expressed by

$$X_{2DResNet}=\overset{n}{\underset{l=0}{\sum }}F\left(X_{l},W_{l}\right)+X_{l},$$ (12)

where *X*_*l*_ is the input feature of the *l*th residual block, being initialized by *X*_0_ = *X*_*2DInput*_, *W*_*l*_ is the weight matrix in the convolutional layers of the *l*th block, *F*() is the mapping function with the same meaning of that in the 1D ResNet block, and *n* is the block number, which is set as 30 in the 2D ResNet module. Hence, the output of the 2D ResNet *X*_*2DResNet*_ will go through the softmax layer and obtain the inter-residue contact label:

$$y=Softmax\left(X_{2DResNet}\right),$$ (13)

where $y\in {\left\{0,1\right\}}^{L\times L}$ , the element *y*_*ij*_ means whether the pair of residue *i* and residue *j* is contacted according to the model (1 for contacted and 0 for uncontacted).

To train the model, the cross-entropy function averaged over all the residue pairs is used as the loss function:

$$E\left(t,y\right)=-\frac{1}{L^{2}}\underset{i}{\sum }\underset{j}{\sum }t_{ij}\log y_{ij},$$ (14)

where *t*_*ij*_ is the true contact label, and *y*_*ij*_ is the predicted contact label between residues *i* and *j*, and *L* is the length of the peptide. For the training process, stochastic gradient descent optimization is utilized, and the learning rate is set as 0.01.

## 3 Results

### 3.1 Training and Test Datasets

In our experiment, we used one training dataset to train our proposed RCMPM model and four different testing datasets to test its performance.

The training dataset is a subset of PDB25 extracted from the PDB database (http://www.rcsb.org/pdb/home/home.do) with homology reduction at 25*%* level of sequence identity, resulting in 6767 non-homologous protein sequences. The number of amino acids of each training protein ranges from 26 to 300. To avoid overfitting, 400 proteins are randomly chosen for validation and the remaining others for training.

To evaluate the performance of our model, it is applied to four testing datasets. The first testing dataset is the PDB25 dataset, which contains 500 nonhomologous protein sequences. The training set, validation dataset, and testing dataset of the abovementioned PDB25 dataset can be downloaded from http://raptorx.uchicago.edu/ContactMap/. The other three datasets were obtained from three CASP (Critical Assessment of Structure Prediction) competitions (CASP10 (Moult et al., 2014), CASP11 (Moult et al., 2016), and CASP12 (Moult et al., 2018)). For the three CASP datasets, we used the same screening method as that used in the R2C method (Yang et al., 2016). For the CASP10 dataset, the sequence data could be accessed on the website of https://predictioncenter.org/download_area/CASP10/targets/. The total number of the sequences is 123. However, seven short sequences are removed (T0651-D3, T0675-D1, T0675-D2, T0677-D1, T0700-D1, T0709-D1, and T0711-D1), and the constructed CASP10 test dataset size is 116. CASP11 and CASP12 datasets are also publicly available on the websites of https://predictioncenter.org/download_area/CASP11/targets/ and https://predictioncenter.org/download_area/CASP12/targets/, with 105 and 55 sizes, respectively. After removing the three short sequences from CASP11 (T0759-D1, T0820-D1, and T0820-D2), the final sizes of CASP11 and CASP12 datasets are 102 and 55, respectively.

### 3.2 Evaluation Metrics

By using the same evaluation criteria as the CASP competition, we evaluated the accuracies of the top *L*/*k* (*k* = 10, 5, 2, 1) predicted contacts, where *L* is the protein sequence length. Accuracy is the proportion of true positive samples in the total number of predicted positive samples, which is defined by

$$Accuracy=\frac{TP}{TP+FP},$$ (15)

where *TP* is the number of predicted contacted pairs being actually contacted, and *FP* is the number of predicted contacted pairs not being actually contacted, respectively. Residue–residue contacts are categorized into three types according to the residue distances in sequence: short-range, medium-range, and long-range corresponding to the distances between 6 and 11, 12 and 23, and at least 24 residues, respectively. It should be noted that a long-range contact places strong constraints on the conformation of peptides and is particularly important for the peptide structure and function study, which is also the main focus of this article.

### 3.3 Performance on PDB25 Testing Datasets and CASP Testing Datasets

In our experiment, we used top *L*/*k* (*k* = 10, 5, 2, 1) in the long-range contact to evaluate the prediction accuracy of contact maps. Here, *L* is the length of the sequence, and the prediction accuracy rates are given in three kinds of contact, namely, long-range, medium-range, and short-range.

The datasets used in our experiment are PDB25, CASP10, CASP11, and CASP12 datasets. To examine the performance of our proposed RCMPM model, three state-of-the-art methods are used for comparison, namely, CCMpred (based on Markov random field pseudo-likelihood maximization, MSA), R2C (based on SVM), and RaptorX-Contact (based on ResNet), respectively. We have realized CCMpred and RaptorX-Contact models and trained them under the same environments with that of the RCMPM and hence obtained the experiment results of the two models by ourselves. The results of R2C on CASP10 and CASP11 datasets are cited from the reference (Villegas-Morcillo et al., 2018), while the results on the other two datasets are calculated through its webserver (http://www.csbio.sjtu.edu.cn/bioinf/R2C/). For comparison, two rough contact maps, produced by CCMpred and RaptorX-Contact models, are used to construct the amino acid graph in the proposed RCMPM, respectively.

**FIGURE 3:**
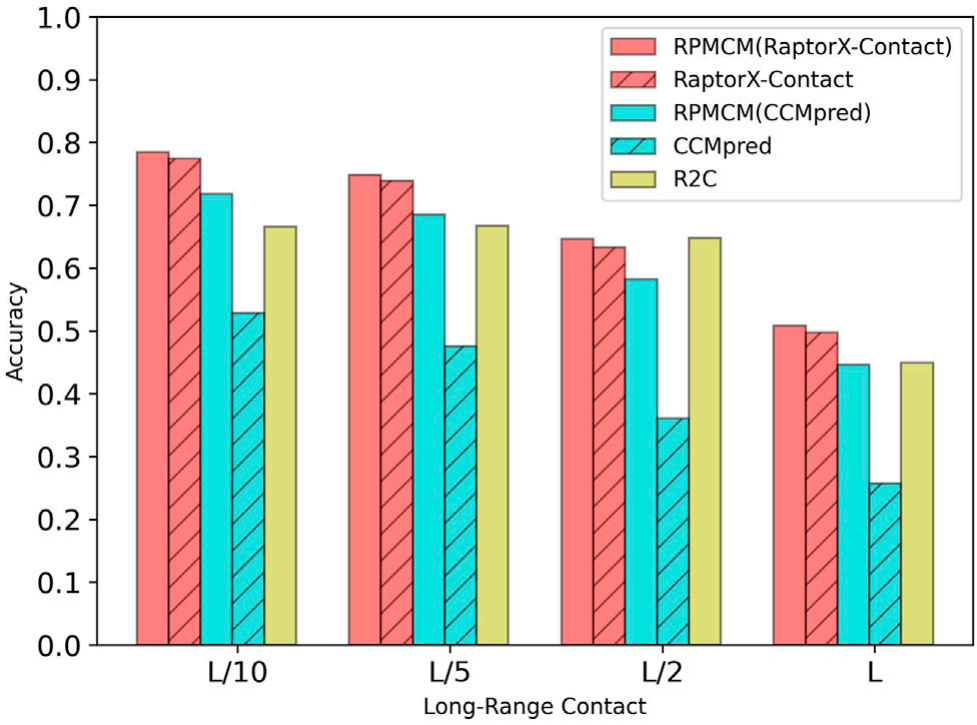
Comparison of method accuracy for the long-range contact on the PDB25 testing dataset.

Table 1 shows the comparison results on the PDB25 dataset. For the long-range contact type prediction, the results of the RCMPM by using the CCMpred outputs as the rough contact map (RCMPM (CCMpred)) are significantly better than those of CCMpred, with 19.9*%*, 22.2*%*, 38.0*%*, and 73.5*%* improvements on top *L*/10, *L*/5, *L*/2, and *L* levels, respectively. Compared to R2C, it improves by 7.8*%* and 2.7*%* on the top *L*/10 and *L*/5 levels, respectively, and decreased by 10.2*%* and 0.7*%* on the top *L*/2 and *L* levels, respectively. RaptorX-Contact is an excellent algorithm, the results of which are better than those of RCMPM (CCMpred). However, when the RCMPM model uses the output of RaptorX-Contact as the rough contact map (RCMPM (RaptorX-Contact)), it outperforms RaptorX-Contact on all the four top levels with 1.3*%*, 1.2*%*, 2.1*%*, and 2.2*%* improvements, respectively. The results of RCMPM (RaptorX-Contact) are also significantly better than CCMpred, R2C, and RCMPM (CCMpred), with the only exception being slightly below R2C at the top *L*/2 level. Figure 3 shows the comparison results of five methods on the long-range contact type prediction. For the medium-range contact type, both RCMPM (CCMpred)) and RCMPM (RaptorX-Contact) are significantly superior to CCMpred and RaptorX-Contact, both outperforming their opponents at the four top levels. Among all the five comparison methods, RCMPM (RaptorX-Contact) performs best at all the four levels. For the short-range contact type, RCMPM (CCMpred)) is greatly better than CCMpred, while RCMPM (RaptorX-Contact) performs similarly with RaptorX-Contact, both significantly outperforming the other three methods.

**TABLE 1:** Contact map results by four different methods on the PDB25 testing dataset.

Method	Long-range				Medium-range				Short-range			
	L/10	L/5	L/2	L	L/10	L/5	L/2	L	L/10	L/5	L/2	L
CCMpred	0.528	0.475	0.361	0.257	0.456	0.356	0.222	0.148	0.356	0.275	0.175	0.121
R2C	0.666	0.667	0.648	0.449	0.591	0.590	0.322	0.176	0.597	0.408	0.201	0.119
RaptorX-Contact	0.774	0.739	0.633	0.497	0.758	0.675	0.469	0.300	0.756	0.641	0.404	0.241
RCMPM (CCMpred)	0.718	0.685	0.582	0.446	0.707	0.622	0.421	0.262	0.685	0.576	0.355	0.208
RCMPM (RaptorX-Contact)	0.784	0.748	0.646	0.508	0.761	0.679	0.473	0.300	0.754	0.645	0.403	0.237

**FIGURE 4:**
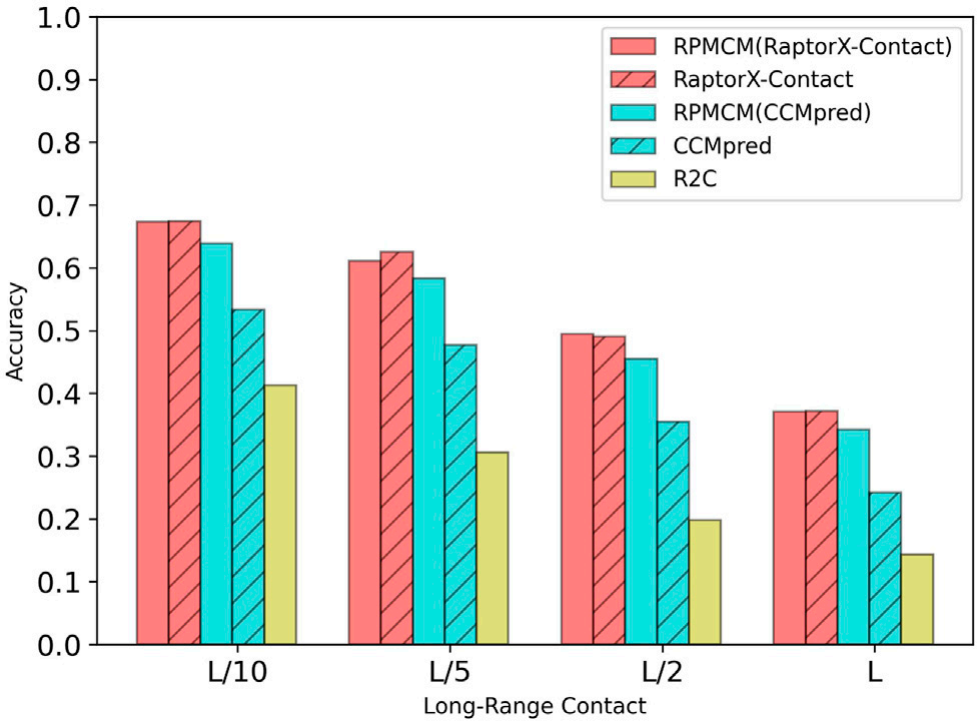
Comparison of method accuracy for the long-range contact on the CASP10 testing dataset.

Table 2 shows the comparison results on the CASP10 dataset. For the long-range contact type prediction, the results of RCMPM by using CCMpred outputs as the rough contact map (RCMPM (CCMpred)) are significantly better than those of CCMpred, with 19.8*%*, 22.2*%*, 28.2*%*, and 41.3*%* improvements on top *L*/10, *L*/5, *L*/2 and *L* levels, respectively. Compared to R2C, it improved by 54.7*%*, 90.5*%*, 129.8*%*, and 139.2*%* at the four top levels, respectively. When the RCMPM uses the RaptorX-Contact outputs as the rough contact map (RCMPM (RaptorX-Contact)), it performs similarly with the RaptorX-Contact, with −0.1*%*, 1.0*%*, −2.2*%* and −0.2*%* variations at the top levels, respectively. Both of them significantly outperform CCMpred and R2C, and a little better than RCMPM (CCMpred). RCMPM (RaptorX-Contact) increases by 26.3*%*, 28.1*%*, 39.4*%*, and 53.3*%* compared to CCMpred and increases by 63.0*%*, 99.7*%*, 150.0*%*, and 159.4*%* compared to R2C at the four top levels, while compared to RCMPM (CCMpred), the improvements are 5.3*%*, 4.8*%*, 8.8*%*, and 8.5*%*, respectively. Figure 4 shows the comparison results of the five methods on the long-range contact type prediction. The results are similar for both the medium-range contact and short-range contact types, with RCMPM (CCMpred) being significantly superior to CCMpred, while RCMPM (RaptorX-Contact), despite its lower performance than RaptorX-Contact, had a weak gap.

**TABLE 2:** Contact map results by four different methods on the CASP10 testing dataset.

Method	Long-range				Medium-range				Short-range			
	L/10	L/5	L/2	L	L/10	L/5	L/2	L	L/10	L/5	L/2	L
CCMpred	0.533	0.477	0.355	0.242	0.512	0.417	0.272	0.185	0.418	0.313	0.197	0.137
R2C	0.413	0.306	0.198	0.143	0.540	0.425	0.278	0.191	0.571	0.511	0.373	0.264
RaptorX-Contact	0.674	0.625	0.490	0.372	0.699	0.629	0.458	0.318	0.638	0.540	0.368	0.233
RCMPM (CCMpred)	0.639	0.583	0.455	0.342	0.646	0.593	0.426	0.290	0.571	0.486	0.316	0.198
RCMPM (RaptorX-Contact)	0.673	0.611	0.495	0.371	0.681	0.612	0.452	0.312	0.630	0.530	0.360	0.225

**FIGURE 5:**
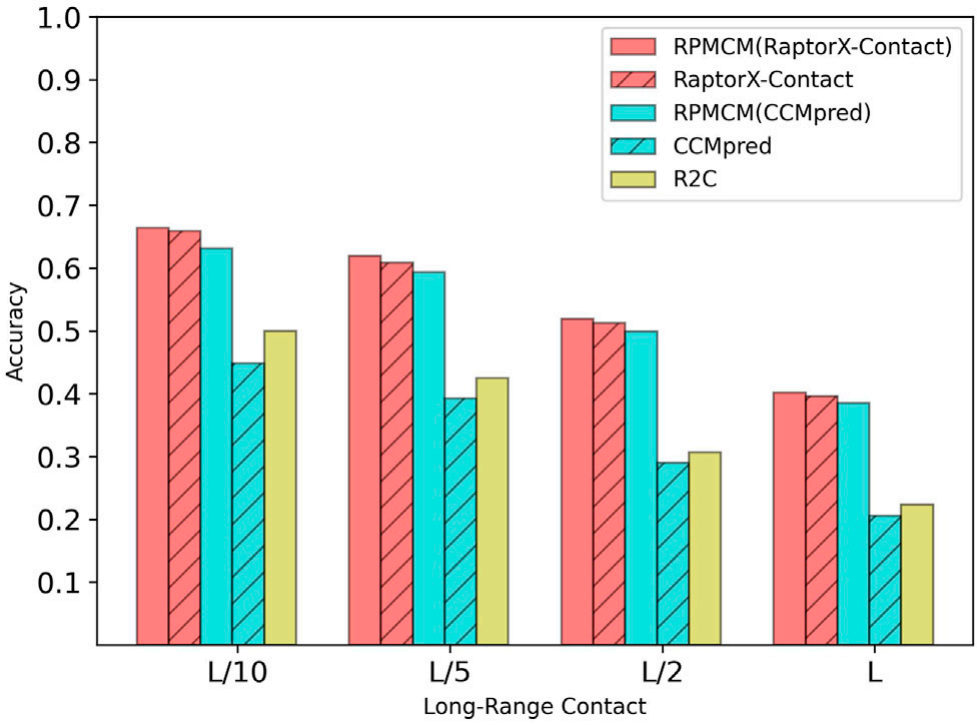
Comparison of method accuracy for the long-range contact on the CASP11 testing dataset.

Table 3 shows the comparison results on the CASP11 dataset. For the long-range contact type prediction, the results of the RCMPM by using the CCMpred outputs as the rough contact map (RCMPM (CCMpred)) are significantly better than those of CCMpred, with 40.8*%*, 50.9*%*, 72.1*%*, and 86.9*%* improvements at the four top levels. Compared to R2C, it outperforms at all the four top levels with 26.2*%*, 39.5*%*, 62.5*%*, and 72.6*%*. RaptorX-Contact is better than RCMPM (CCMpred). However, when the RCMPM uses the output of RaptorX-Contact as the rough contact map (RCMPM (RaptorX-Contact)), it improves by 0.8*%*, 1.8*%*, 1.4*%*, and 1.5*%* on the four top levels, respectively. The results of RCMPM (RaptorX-Contact) are also significantly better than CCMpred and R2C. The results are similar for both the medium-range contact and short-range contact types, with RCMPM (CCMpred) being significantly superior to CCMpred, while RCMPM (RaptorX-Contact), despite its lower performance than RaptorX-Contact, had a weak gap. Figure 5 shows the comparison results of the five methods on the long-range contact type prediction. For both the medium-range contact and short-range contact types’ results, we can draw the following conclusions: among the three existing state-of-the-art (SOTA) methods, RaptorX-Contact performs the best; RCMPM (CCMpred) is significantly superior to CCMpred; RCMPM (RaptorX-Contact) obtains similar results with RaptorX-Contact, while CCMpred is much lower than RaptorX-Contact; and RCMPM (CCMpred) has yielded results comparable to RCMPM (RaptorX-Contact).

**TABLE 3:** Contact map results by four different methods on the CASP11 testing dataset.

	Long-range				Medium-range				Short-range			
Method	L/10	L/5	L/2	L	L/10	L/5	L/2	L	L/10	L/5	L/2	L
CCMpred	0.448	0.393	0.290	0.206	0.376	0.298	0.187	0.132	0.318	0.251	0.162	0.118
R2C	0.500	0.425	0.307	0.223	0.397	0.296	0.192	0.138	0.314	0.228	0.146	0.115
RaptorX	0.659	0.608	0.512	0.396	0.677	0.608	0.447	0.296	0.683	0.598	0.405	0.249
RCMPM (CCMpred)	0.631	0.593	0.499	0.385	0.644	0.593	0.431	0.277	0.646	0.577	0.380	0.224
RCMPM (RaptorX-Contact)	0.664	0.619	0.519	0.402	0.670	0.608	0.450	0.299	0.682	0.601	0.406	0.245

**FIGURE 6:**
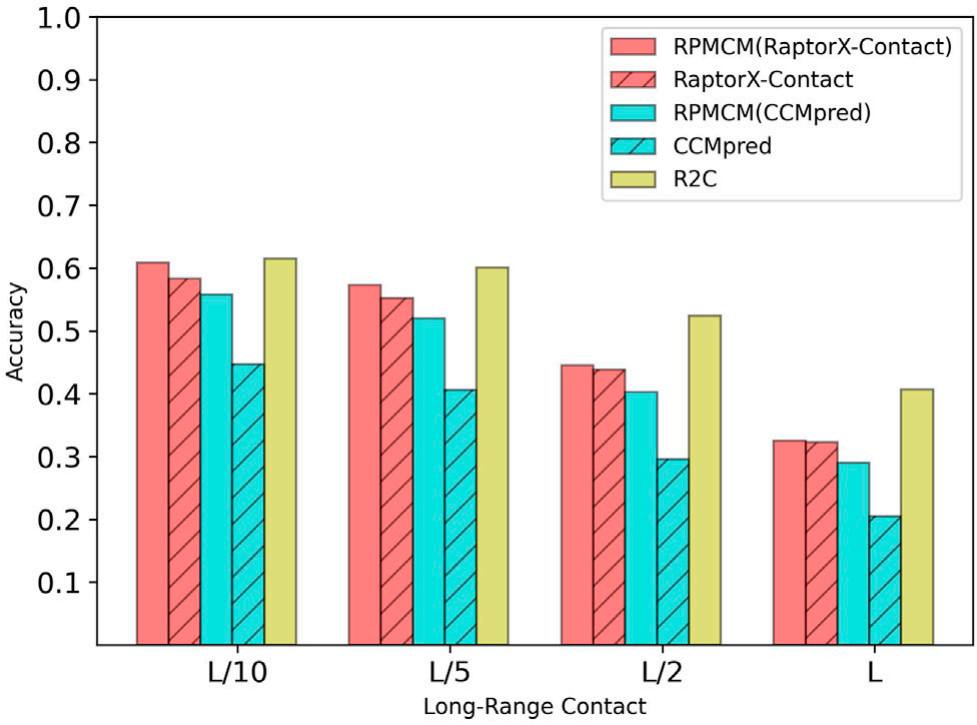
Comparison of method accuracy for the long-range contact on the CASP12 testing dataset.

Table 4 shows the comparison results on the CASP12 dataset. For the long-range contact type prediction, the results of the RCMPM by RCMPM (CCMpred) are significantly better than those of CCMpred, with 24.8*%*, 28.1*%*, 36.1*%*, and 41.5*%* improvements at the top L/10, L/5, L/2, and L levels, while RCMPM (RaptorX-Contact) outperforms RaptorX-Contact with 4.3*%*, 3.8*%*, 1.6*%*, and 0.6*%* improvements on the four top levels, respectively. The results of RCMPM (RaptorX-Contact) are also significantly better than those of CCMpred and RCMPM (CCMpred). Figure 6 shows the comparison results of the five methods on the long-range contact type prediction. For both the medium-range contact and short-range contact types’ results, we can draw the following conclusions: among the three existing SOTA methods, R2C performs the best; RCMPM (CCMpred) is significantly superior to CCMpred; RCMPM (RaptorX-Contact) obtains similar results with RaptorX-Contact; CCMpred is much lower than RaptorX-Contact, but the gap between RCMPM (CCMpred) and RCMPM (RaptorX-Contact) is greatly reduced.

**TABLE 4:** Contact map results by four different methods on the CASP12 testing dataset.

Method	Long-range				Medium-range				Short-range			
	L/10	L/5	L/2	L	L/10	L/5	L/2	L	L/10	L/5	L/2	L
CCMpred	0.447	0.406	0.296	0.205	0.421	0.339	0.205	0.136	0.355	0.256	0.165	0.119
R2C	0.615	0.601	0.524	0.407	0.622	0.545	0.399	0.259	0.584	0.502	0.323	0.205
RaptorX	0.583	0.552	0.438	0.323	0.616	0.545	0.371	0.247	0.581	0.488	0.331	0.222
RCMPM (CCMpred)	0.558	0.520	0.403	0.290	0.586	0.492	0.329	0.213	0.525	0.438	0.278	0.177
RCMPM (RaptorX-Contact)	0.608	0.573	0.445	0.325	0.606	0.530	0.372	0.245	0.591	0.484	0.333	0.215

To summarize the long-range results of the four datasets, it could be found that our proposed RCMPM method is significantly superior to the other methods on PDB25 and CASP11. For CASP10, RCMPM performs much better than CCMpred and R2C, and although it does not perform as well as RaptorX, the gap is very small. For CASP12, the accuracy of RCMPM is higher than that of CCMpred and RaptorX, and is slightly lower than that of R2C. Therefore, it can be concluded that our proposed method performs best overall on the four datasets and is the most stable one as well.

### 3.4 Ablation Study

#### 3.4.1 Evaluation of the GCN Module of the Model Structure

In order to examine the effectiveness of our proposed method, we used two network structures to construct different network structures, the original RCMPM and the RCMPM removal GCN module (RCMPM (without GCN)). Table 5 shows the comparison results on the PDB25 dataset. Compared to the RCMPM (without GCN), the RCMPM improves by 1.2*%*, 0.9*%*, 1.7*%*, and 2*%* on the top *L*/10, *L*/5, *L*/2, and *L* levels, respectively, while the RCMPM performs much similar with the RCMPM (without GCN) on both the short-range and medium-range levels. This is because the graph neural network module can utilize the output of the existing methods, especially on the global information level, and therefore reflected by improvements on the long-range level contact prediction.

**TABLE 5:** Contact map results by the comparison between our network structures.

Method	Long-range				Medium-range				Short-range			
	L/10	L/5	L/2	L	L/10	L/5	L/2	L	L/10	L/5	L/2	L
RCMPM (without GCN)	0.775	0.741	0.635	0.498	0.761	0.676	0.471	0.299	0.755	0.642	0.402	0.238
RCMPM	0.784	0.748	0.646	0.508	0.761	0.679	0.473	0.300	0.754	0.645	0.403	0.237

#### 3.4.2 Evaluation of Different Feature Combinations

In order to verify the effectiveness of the sequence features on the long-range contact map prediction, we used three different feature combinations as the input of the 1D ResNet module and GCN module, including PSSM, PSSM, and secondary structure (PSSM+SS), including the PSSM, secondary structure, and solvent accessibility (PSSM+SS+SA), a total of L×26 dimensional features. Table 6 shows the comparison results by using the RaptorX-Contact outputs as the rough contact map on the PDB25 dataset. From Table 6, it could be found that on the long-range contact type, RCMPM (PSSM+SS+SA) is improved by 0.9*%*, 0.8*%*, 1.7*%*, and 0.6*%* at the four top levels compared to RCMPM (PSSM+SS) and 1.6*%*, 0.9*%*, 1.1*%*, and 1.2*%* on the four top levels compared to RCMPM (PSSM). Meanwhile, on the medium-range contact type, although the trend is the same as the long-range type, the increase is very small. On the short-range contact type, the results of the three methods are even very close. Table 7 shows the comparison results by using the CCMpred outputs as the rough contact map on the PDB25 dataset. From Table 7, it could be found that on the long-range contact type, RCMPM (PSSM+SS+SA) is improved by 0.8*%*, 2.2*%*, 2.1*%*, and 2.1*%* at the four top levels compared to RCMPM (PSSM+SS) and 9.3*%*, 13.4*%*, 26.2*%*, and 44.8*%* at the four top levels compared to RCMPM (PSSM). On the medium-range and short-range contact types, the results of the RCMPM (PSSM+SS+SA) are also better than the other two types’ results. The results show that the PSSM is a very important feature for the contact prediction, and the secondary structure and solvent accessibility are also beneficial. When the initial contact map is used as RaptorX-Contact, the secondary structure and solvent accessibility have a limited effect on the medium- and short-range contact type predictions, in part because the RCMPM uses the output of RaptorX-Contact, which already contains the secondary structure and solvent accessibility information.

**TABLE 6:** Comparison results for feature combinations by using the rough RaptorX-Contact contact map.

Method	Long-range				Medium-range				Short-range			
	L/10	L/5	L/2	L	L/10	L/5	L/2	L	L/10	L/5	L/2	L
RCMPM (PSSM)	0.772	0.741	0.639	0.502	0.760	0.674	0.467	0.297	0.761	0.642	0.403	0.238
RCMPM (PSSM+SS)	0.777	0.742	0.639	0.505	0.760	0.673	0.469	0.298	0.756	0.643	0.401	0.237
RCMPM (PSSM+SS+SA)	0.784	0.748	0.646	0.508	0.761	0.679	0.473	0.300	0.754	0.645	0.403	0.237

**TABLE 7:** Comparison results for feature combinations by using the rough CCMpred contact map.

Method	Long-range				Medium-range				Short-range			
	L/10	L/5	L/2	L	L/10	L/5	L/2	L	L/10	L/5	L/2	L
RCMPM (PSSM)	0.657	0.604	0.461	0.308	0.614	0.499	0.299	0.180	0.581	0.438	0.238	0.138
RCMPM (PSSM+SS)	0.712	0.670	0.570	0.437	0.692	0.609	0.411	0.254	0.680	0.569	0.346	0.201
RCMPM (PSSM+SS+SA)	0.718	0.685	0.582	0.446	0.707	0.622	0.421	0.262	0.685	0.576	0.355	0.208

## 4 Discussion

In this article, we formulated the peptide contact map refinement task as a geometric 2D graph improvement and proposed a novel refined contact map prediction model (RCMPM) to refine the protein inter-residue contact map predictions using graph convolutional neural networks (GCNNs) and one-dimensional and two-dimensional residual neural network (1D ResNet and 2D ResNet) architectures. Our method combines the residual neural networks for learning the local information with the graph convolutional neural networks for learning the global information, which can better capture the long-range contact relationship between the complex sequence inter-residues. The experimental results show that our method can refine the contact map greatly for the long-range contact type, that is to say, by using CCMpred outputs as the rough contact map, the RCMPM is significantly better than CCMpred, and by using the RaptorX-Contact outputs as the rough contact map, the RCMPM is significantly better than RaptorX-Contact as well. For the medium-range contact prediction, the degree of improvement is significantly reduced, and for the short-range contact prediction, there is not even a significant improvement. The main reason is that the GCN module of the RCMPM can utilize the outputs of the existing methods, which are highly reflected on the global information level, and therefore, the RCMPM model makes improvements mainly on the long-range contact types. By using a larger protein database in HHblits or PSI-BLAST to calculate the homology features of protein sequences and combining more effective features as inputs, we can expect to further improve the precision.

## Data Availability

The original contributions presented in the study are included in the article/Supplementary Material, further inquiries can be directed to the corresponding author.
